# Supplementary figures and images for: Response Gene to Complement 32 promotes cell proliferation and tamoxifen resistance in breast cancer via elevated FoxM1 expression
Source: PLoS One. 2025 Jul 28;20(7):e0328698. doi: 10.1371/journal.pone.0328698 (PMC12303305; doi:10.1371/journal.pone.0328698)

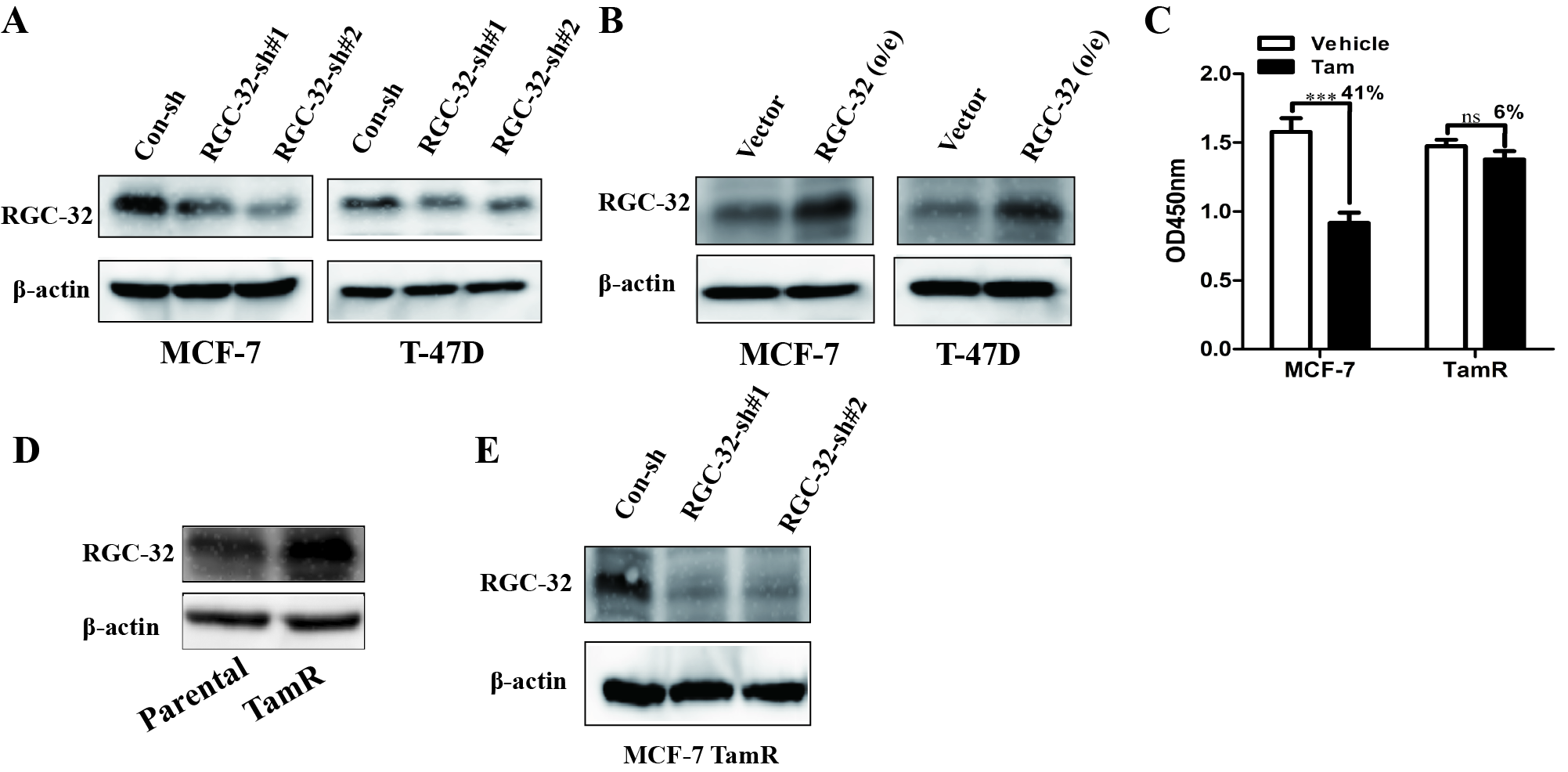

Supplement: S1 Fig — A-B, The expression levels of RGC-32 in breast cancer cells stably transfected with RGC-32 shRNA and control shRNA or plasmids containing RGC-32 and the empty vector, were determined by western-blotting. C, Parental and tamoxifen resistant (TamR) cells were treated with 1µM tamoxifen or vehicle for 5 days. Cell viability was assessed by CCK8 assay. D, The expression levels of RGC-32 in parental and TamR cells. E, The expression levels of RGC-32 in TamR cells stably transfected with RGC-32 shRNA and control shRNA were determined by western-blotting. (TIF) [file pone.0328698.s001.tif]

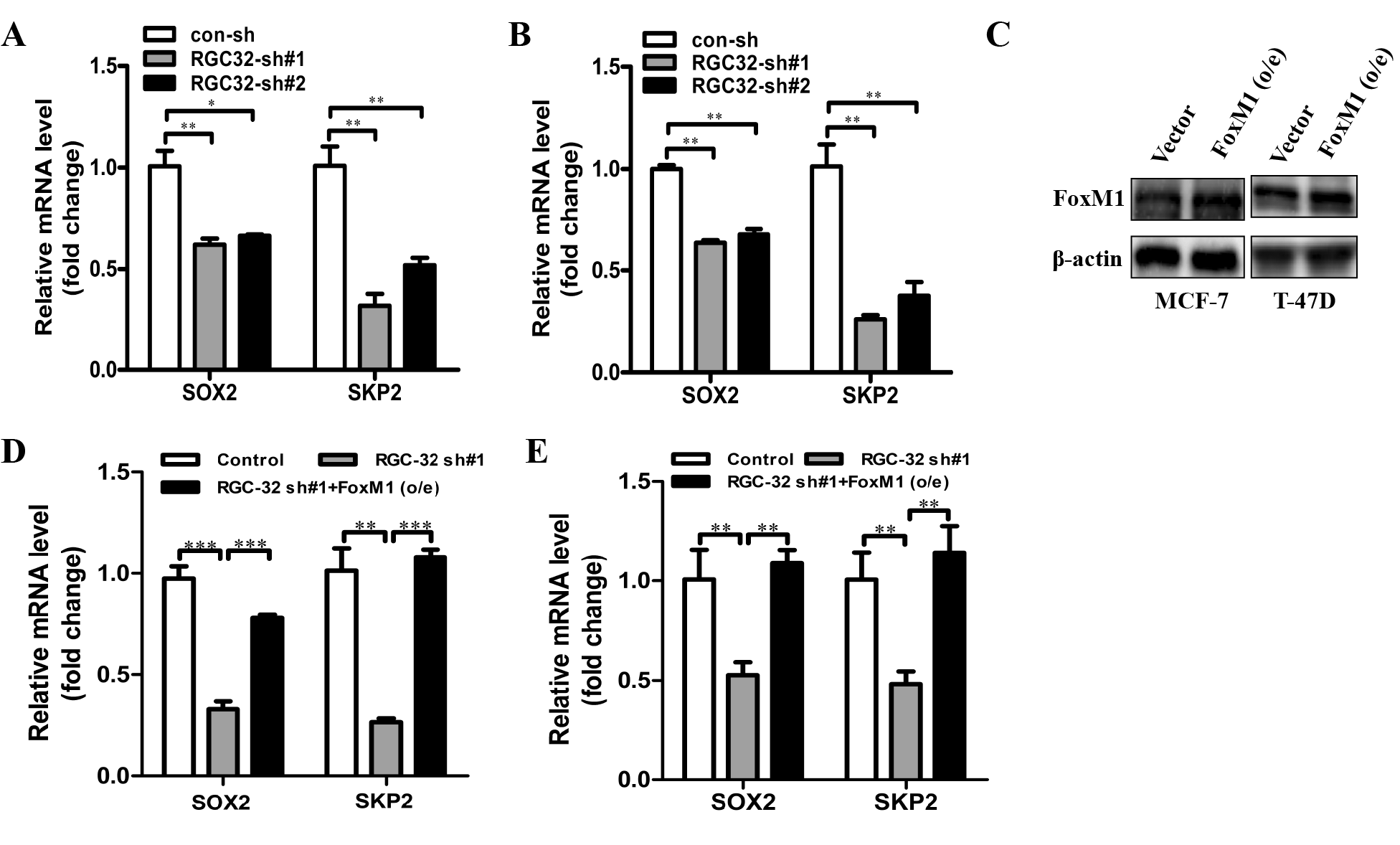

Supplement: S2 Fig — A-B, RGC-32 knockdown decreased SKP2 and SOX2 mRNA expression in MCF-7 and T-47D cells. *P < 0.05; **P < 0.01; ***P < 0.001 for target gene expression comparison. C, Breast cancer cells were transfected with plasmids expressing FoxM1. The expression levels of FoxM1 were determined by western-blotting. D, Breast cancer cells were co-transfected with plasmids expressing FoxM1, RGC-32-shRNA or empty vector. SKP2 and SOX2 mRNA expression were determined by Q-PCR. (TIF) [file pone.0328698.s002.tif]

**Fig 4D**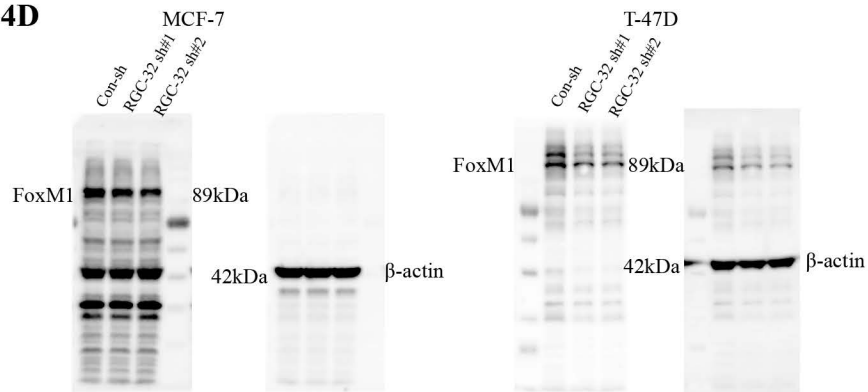**Fig 5A**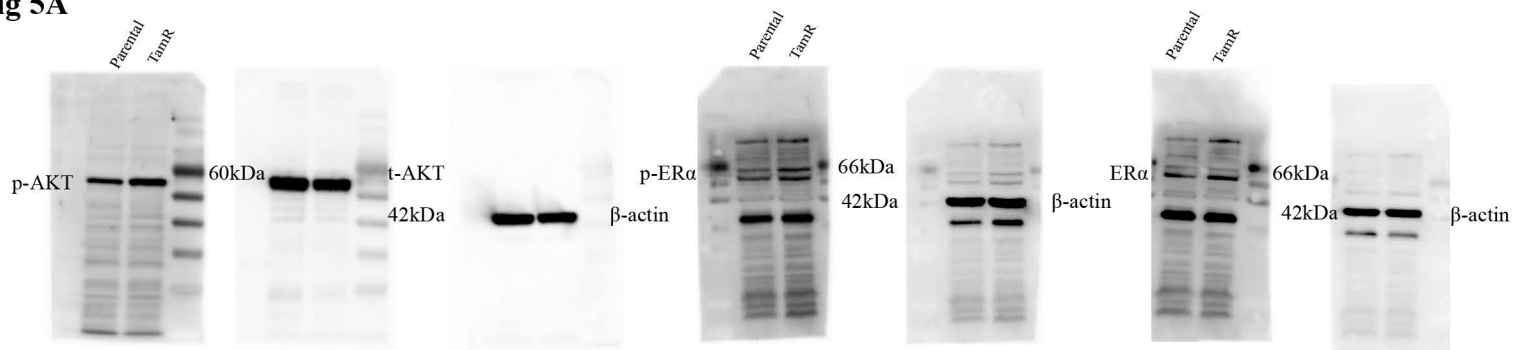**Fig 5B**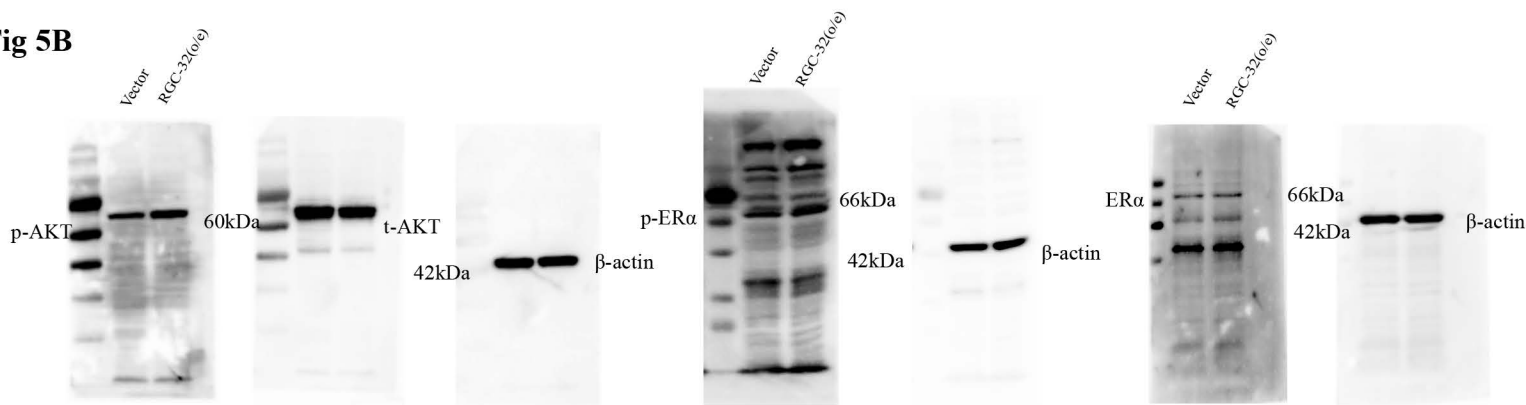**Fig 5C**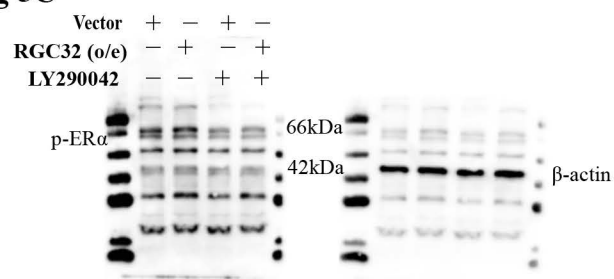**Fig 5D**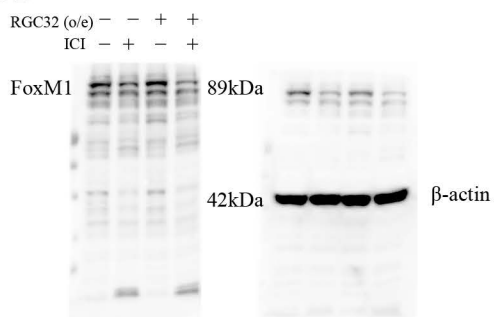**Fig 5E**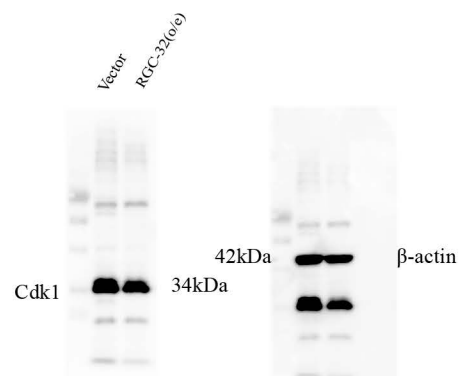

**Fig S5G**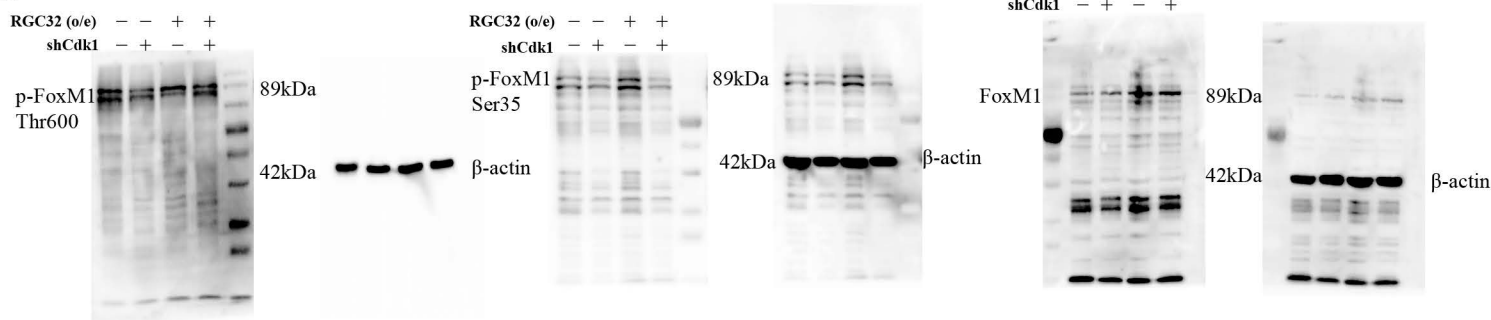**Fig S1A**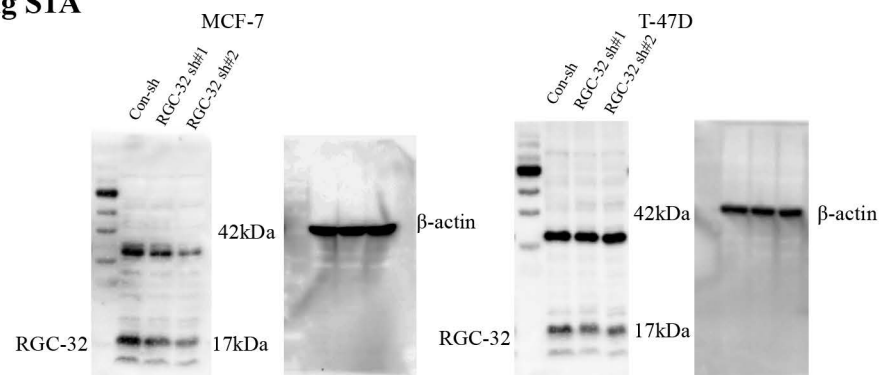**Fig S1B**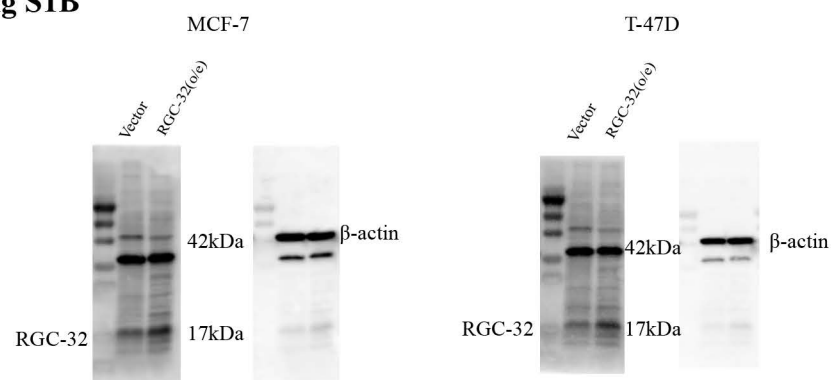**Fig S1D**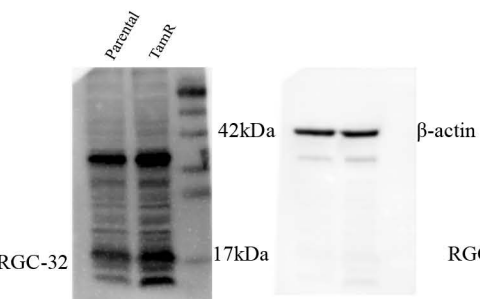**Fig S1E**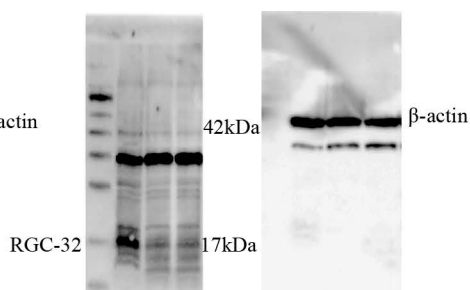**Fig S2C**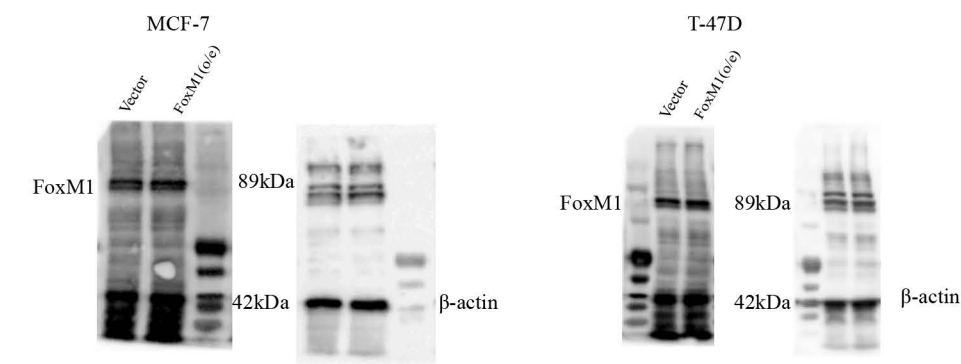

Supplement: S1 File — (PDF) [file pone.0328698.s004.pdf]
