# Supplementary material for: Response Gene to Complement 32 promotes cell proliferation and tamoxifen resistance in breast cancer via elevated FoxM1 expression
Source: PLoS One. 2025 Jul 28;20(7):e0328698. doi: 10.1371/journal.pone.0328698 (PMC12303305; doi:10.1371/journal.pone.0328698)
Supplement: S1 Table — (DOCX) [file pone.0328698.s003.docx]

| **shRNAs** | **Sense Strand (5’-3’)** | |
| --- | --- | --- |
| shRGC32#1 | CACTCCTCAGAAAGCTAAA | |
| shRGC32#2 | ACAGACGATCCATGCTAA | |
| shControl | TTCTCCGAACGTGTCACGT | |
| ShCDK1 | GGCACTGAATCATCCATATTT | |
| Negative Control | TTCTCCGAACGTGTCACGT | |
| **Primers for qRT-PCR** | **Sense Strand (5'-3')** | **Antisense Strand (3'-5')** |
| SKP2 | GCTGCTAAAGGTCTCTGGTGT | AGGCTTAGATTCTGCAACTTG |
| SOX2 | CCCTGTGGTTACCTTTTCCT | AGTGCTGGGACATGTGAAGT |

**Supplementary Table S1. Sequences of the shRNAs and primers for ChIP assays and qRT-PCR analysis.**
